# Evaluation of the HbA1c Reduction Cut Point for a Nonglycemic Effect on Cardiovascular Benefit of Hypoglycemic Agents in Patients with Type 2 Diabetes Based on Endpoint Events

**DOI:** 10.1155/2018/8457538

**Published:** 2018-04-19

**Authors:** Yuchao Wu, Lizhi Tang, Fang Zhang, Zhe Yan, Jing Li, Nanwei Tong

**Affiliations:** Department of Endocrinology and Metabolism, West China Hospital, Sichuan University, Chengdu, China

## Abstract

**Background:**

Atherosclerotic cardiovascular disease (ASCVD) is a major cause of death among patients with diabetes but can be improved by certain hypoglycemic agents. However, adjudicating criteria on whether improvements are a glycemic or nonglycemic effect of these agents remain unclear.

**Methods:**

Hypoglycemic agents that produce a cardiovascular benefit in nondiabetic patients are considered to do so via a nonglycemic effect. We performed a subgroup analysis for primary and secondary prevention or very high risk of ASCVD in patients with type 2 diabetes (T2DM). Where glycosylated hemoglobin (HbA1c) was reduced to the same extent in a head-to-head comparison, cardiovascular benefits were judged as a nonglycemic effect. Furthermore, by analyzing the endpoints of four important randomized controlled intensive glucose control studies, UKPDS33, ADVANCE, ACCORD, and VADT, we calculated the cut point of HbA1c reduction for a nonglycemic effect on cardiovascular benefit by hypoglycemic agents in ASCVD groups of different severities.

**Results:**

For the ASCVD primary prevention group of T2DM, UKPDS33 indicated a reduction in HbA1c < 0.9%, and a cardiovascular benefit within 10 years was considered a nonglycemic effect. For ASCVD secondary prevention or in the very high-risk group, pioglitazone exerted a nonglycemic effect on cardiovascular benefit in nondiabetic patients with insulin resistance; metformin may exert a similar effect in T2DM patients in a head-to-head study. Analysis of T2DM intensive glucose control studies showed a reduction in HbA1c of <1.0%, and a cardiovascular benefit after approximately 5 years was deemed a nonglycemic effect.

**Conclusions:**

For ASCVD primary prevention in T2DM, a reduction in HbA1c < 0.9% and a cardiovascular benefit within 10 years were considered a nonglycemic effect. For ASCVD secondary prevention or in a very high-risk population, a reduction in HbA1c < 1.0% and a cardiovascular benefit within about 5 years were also considered a nonglycemic effect.

## 1. Background

With the development of society and changes in lifestyle, the global incidence of diabetes and expenditure on the disease are constantly increasing. The global estimate of adults with diabetes was 415 million in 2015 or one in 11 adults. This number is expected to rise beyond 642 million by 2040, when one in ten adults will have diabetes [1]. Atherosclerotic cardiovascular disease (ASCVD) is the leading cause of death in diabetic patients, with approximately 70% of deaths associated with ASCVD in this population [2]. Type 2 diabetes (T2DM) is an important independent risk factor for ASCVD, with hospital mortality and rate of cardiovascular disease (CVD) twofold that of patients without diabetes [3, 4]. Oral hypoglycemic agents exert different effects on CVD outcome. For example, the IRIS trial included patients without diabetes who had insulin resistance and a recent history of cerebrovascular disease and showed that pioglitazone had a significant cardiovascular benefit [5]. Trials including DPP-4 inhibitors such as EXAMINE (alogliptin), SAVOR-TIMI 53 (saxagliptin), and TECOS (sitagliptin) [6–8], and GLP-1 receptor agonists trials such as ELIXA (lixisenatide) [9], did not show a significant decrease in ASCVD morbidity and all-cause mortality compared with control. However, the GLP-1 receptor agonist trials LEADER (liraglutide) and SUSTAIN-6 (semaglutide) [10, 11] and the SGLT2 inhibitor trial EMPA-REG (empagliflozin) [12] showed a reduction in glycosylated hemoglobin (HbA1c) similar to that of the above trials. However, although the patient populations were similar across these trials, LEADER, EMPA-REG, and SUSTAIN-6 all showed a significant decrease in ASCVD, indicating that the cardiovascular benefit shown in these three trials may not be a glycemic effect. Where patient populations and reductions in HbA1c are similar, it is clear that some hypoglycemic agents have a cardiovascular benefit but others do not, indicating that the cardiovascular benefit is a nonglycemic effect independent of HbA1c reduction. However, there is no consensus on the cut point of HbA1c reduction with a simultaneous cardiovascular benefit that can be judged as a nonglycemic effect. To determine the HbA1c cut point, nondiabetic patients should theoretically be included in interventions, as in the IRIS trial [5], although this may not be entirely feasible. Head-to-head comparisons of studies reported in the literature may also be used to compare the cardiovascular benefit of hypoglycemic agents with the same reduction in HbA1c. In the present study, we sought to determine a specific cut point in HbA1c reduction in T2DM patients based on CVD endpoints by analyzing data from specific landmark intensive glucose control studies. When HbA1c decreases below this cut point and a cardiovascular benefit is observed, the hypoglycemic agents can be considered to exert a nonglycemic effect on cardiovascular benefits in T2DM patients.

## 2. ASCVD Stratification of Patients in Published Large-Scale Studies

There is grading of ASCVD prevention in diabetic patients. In this study, primary prevention of ASCVD was defined as the prevention of ASCVD in T2DM patients without clinical ASCVD. Secondary prevention of ASCVD was defined as the prevention of established clinical ASCVD from recurrence, reducing mortality, and improving quality of life in patients with T2DM. Patients with diabetes were considered at high risk of ASCVD, and diabetic patients with other risk factors (hypertension, smoking, dyslipidemia, etc.) for ASCVD were considered at very high risk of ASCVD. Since the patients included in the majority of the known intensive glucose control studies on T2DM had few independent ASCVD secondary prevention groups, and secondary prevention for ASCVD and very high risk of ASCVD were often included in the same group, the data for these two populations could not be distinguished in the studies. Thus, we pooled these patients as one group and described it as secondary prevention or very high risk of ASCVD. In addition, specific preventive measures for ASCVD and control target values for blood glucose, blood pressure, or other indicators were also different between the ASCVD primary prevention group and the ASCVD secondary prevention or very high-risk group; therefore, we divided the enrolled participants into an ASCVD primary prevention group and an ASCVD secondary prevention or very high-risk group to evaluate the nonglycemic effects of hypoglycemic agents on cardiovascular benefit.

## 3. Evidence from the Studies in Nondiabetic Patients

The IRIS study included 3867 patients with insulin resistance and a recent history of cerebrovascular disease but without diabetes, and the primary outcome was fatal or nonfatal stroke or myocardial infarction. By 4.8 years, the primary outcome was significantly reduced in the pioglitazone group compared with the control group (hazard ratio (HR) 0.76, 95% confidence interval (CI) 0.62–0.93, *p* = 0.007); that is, pioglitazone exerted a significant cardiovascular benefit in this patient population independent of its glycemic effect [5].

The STOP-NIDDM study included 1429 patients with impaired glucose tolerance (IGT), and the main outcome was development of major cardiovascular events (coronary heart disease, cardiovascular death, congestive heart failure, cerebrovascular events, and peripheral vascular disease) and hypertension (≥140/90 mmHg). After 3.3 years of follow-up, acarbose treatment was associated with a 49% relative risk reduction in the development of cardiovascular events compared with the control group (HR 0.51, 95% CI 0.28–0.95, *p* = 0.03) [13]. However, the patient population in this study was relatively small, with few events and a high rate of patients lost to follow-up, so the nonglycemic effect of acarbose on cardiovascular benefit in IGT patients can only be inferred.

The DPP study included 3234 IGT patients randomly assigned to receive intensive lifestyle intervention, metformin, or placebo. Annual assessment of blood pressure, lipids, electrocardiogram, and CVD events was performed. After 3 years, the use of pharmacologic therapy to achieve established goals in the intensive lifestyle group was 27-28% less for hypertension and 25% less for hyperlipidemia compared with the placebo and metformin groups (*p* < 0.001). However, neither the cumulative incidence of CVD nor the event rate was different between the groups. This study showed that lifestyle intervention improves CVD risk factor status compared with placebo and metformin therapy. However, metformin did not exert a significant cardiovascular benefit in IGT/prediabetic patients [14].

## 4. Evidence from a Head-to-Head Study

In a head-to-head study of 304 T2DM patients with coronary artery disease (CAD), the primary endpoints were time to the composite of recurrent cardiovascular events, including death from a cardiovascular cause, death from any cause, nonfatal myocardial infarction, nonfatal stroke, or arterial revascularization. Treatment with metformin for 3 years substantially reduced major cardiovascular events in a median follow-up period of 5 years compared with glipizide (HR 0.54, 95% CI 0.30–0.90, *p* = 0.026), although glucose control was similar between these two groups (HbA1c was 7.1% in the glipizide group and 7.0% in the metformin group) [15]. Given the small size of this study, we could only infer that metformin may have exerted a nonglycemic effect on cardiovascular benefit in these patients.

## 5. Analysis and Conclusion of the Cut Point of HbA1c Reduction Rate of Nonglycemic Effect in T2DM Patients

In recent years, increasing numbers of glucose control studies have been reported. We selected landmark intensive glucose control studies to analyze and interpret in this study, including randomized controlled trials in which the hypoglycemic effect influenced CVD in T2DM patients with a large sample size (≥1000 patients) and a follow-up time of ≥3 years. Data from the UKPDS33, ADVANCE, ACCORD, and VADT studies were analyzed to identify the specific cut point of HbA1c reduction that had a nonglycemic beneficial effect in two groups: (1) ASCVD primary prevention and (2) ASCVD secondary prevention or very high risk.

### 5.1. Primary ASCVD Prevention Group

#### 5.1.1. Cut Point Analysis

As a large population is required to assess the nonglycemic effect on cardiovascular benefit in an ASCVD primary prevention group, we examined data from the UKPDS33 study. This study included 3867 newly diagnosed patients with T2DM whose median age was 54 years and who were randomly assigned to an intensive glucose control group (sulfonylurea or insulin) or conventional policy group with diet (diet alone; drugs were added only for hyperglycemic symptoms or FPG (fasting plasma glucose) > 15 mmol/L). The endpoints were any diabetes-related event (sudden death, death from hyperglycemia or hypoglycemia, fatal or nonfatal myocardial, stroke, etc.), diabetes-related death (death from myocardial infarction, stroke, peripheral vascular disease, etc.), and all-cause mortality. After a median of 10 years follow-up, HbA1c was 7.0% (6.2–8.2) in the intensive group compared with 7.9% (6.9–8.8) in the conventional group. Patients in the intensive group had a 6% reduction in all-cause mortality (−10 to 20, *p* = 0.44) and a 16% reduction in myocardial infarction (0 to 29, *p* = 0.052) but no difference in silent myocardial infarction, cardiomegaly, or peripheral vascular disease compared with the conventional group [16]. This outcome indicated that intensive glucose control did not reduce morbidity from macrovascular events in T2DM, while the reduction in myocardial infarction events was of borderline significance.

Although there was only one UKPDS33 study, the sample size of this study was large, follow-up time was 10 years, and it was recognized as one of the major intensive glucose control studies by endocrine academia. Therefore, we can roughly postulate a cut point of nonglycemic effect of cardiovascular benefit in the ASCVD primary prevention group through the analysis of the results. According to the results of the UKPDS33, macrovascular benefit of intensive glucose control was not obvious for the ASCVD primary prevention group, while the reduction of myocardial infarction was at borderline significance. Mean HbA1c in the intensive glucose control group was 0.9% lower than that in the conventional group by the end of the study. From this, we inferred that the risk of myocardial infarction may decrease in the ASCVD primary prevention group after 10 years when HbA1c is reduced by at least 0.9%. Thus, if the reduction in HbA1c in the experimental group was <0.9% compared with control in hypoglycemic agent trials, the relevant ASCVD (i.e., macrovascular events) benefit should represent a nonglycemic effect. We therefore selected a reduction in HbA1c of <0.9% as a specific cut point to assess the nonglycemic effect of cardiovascular benefit in the ASCVD primary prevention group. If HbA1c was reduced <0.9% and a cardiovascular benefit was observed within 10 years, it can be considered a nonglycemic effect.

#### 5.1.2. Cut Point Verification

The UKPDS34 study included 753 overweight patients with T2DM in an ASCVD primary prevention group. The primary outcomes were any diabetes-related clinical endpoint, diabetes-related death, and all-cause mortality. After 10.7 years of follow-up, the metformin group had a 36% lower risk (*p* = 0.011) of all-cause mortality, a 39% lower risk (*p* = 0.010) of myocardial infarction, and a 30% (*p* = 0.020) lower risk of all macrovascular diseases together (myocardial infarction, sudden death, angina, stroke, and peripheral disease) than the conventional treatment group. HbA1c was reduced by 0.6% in the metformin group compared with the conventional group at the end of the study [17]. This result was obtained in a subgroup analysis and may therefore lack sufficient evidence to show that metformin exerted a nonglycemic effect on overweight patients with T2DM. Some related studies have reported that nonglycemic effects of metformin on cardiovascular benefit may be attributed to weight loss and antiatherosclerosis effects [18–20].

#### 5.1.3. Conclusion

The UKPDS33 study reported a reduction in HbA1c of <0.9%, and if a cardiovascular benefit was observed within 10 years, the benefit was a nonglycemic effect. Metformin may therefore exert a nonglycemic effect on cardiovascular benefit in overweight patients with T2DM according to this cut point.

### 5.2. ASCVD Secondary Prevention or Very High-Risk Group

#### 5.2.1. Cut Point Analysis

We evaluated the ADVANCE, ACCORD, and VADT intensive glucose control studies to determine the cut point of HbA1c reduction for a nonglycemic effect on ASCVD benefit in an ASCVD secondary prevention or very high-risk group. The ADVANCE study included 11,140 T2DM patients of median age 66 years with a history of macrovascular events or at least one other risk factor for CVD, randomly assigned to intensive (gliclazide plus other drugs) or standard (sulfonylurea but not gliclazide) glucose control. The primary endpoints were major macrovascular events and major microvascular events. After a median of 5 years of follow-up, mean HbA1c was 6.5% in the intensive glucose control group and 7.3% in the standard control group, a difference of 0.8%. No significant difference was observed between the groups for major macrovascular events (HR 0.94, 95% CI 0.84–1.06, *p* = 0.32), death from cardiovascular causes (HR 0.88, 95% CI 0.74–1.04, *p* = 0.12), or death from any cause (HR 0.93, 95% CI 0.83–1.06, *p* = 0.28) [21]. The ACCORD study included 10,251 T2DM patients (mean age, 62.2 years) with a history of cardiovascular events (35% of total patients) or other CVD risk factors and randomly assigned to an intensive therapy or standard therapy group with no special restriction on hypoglycemic agents. The primary outcomes were nonfatal myocardial infarction, nonfatal stroke, or death from cardiovascular causes. After a mean of 3.5 years of follow-up, median HbA1c was 6.4% in the intensive therapy group and 7.5% in the control group, a difference of 1.1%. There was no significant difference between the groups on primary outcomes (HR 0.90, 95% CI 0.78–1.04, *p* = 0.16), although mortality was increased in the intensive therapy group (HR 1.22, 95% CI 1.01–1.40, *p* = 0.04) [22]. The VADT study included 1791 T2DM patients with a mean age of 60.4 years and a suboptimal response to therapy (40% of patients had a history of cardiovascular events), randomly assigned to an intensive or a standard glucose control group (patients with BMI ≥ 27 were started on metformin plus rosiglitazone; those with BMI < 27 were started on glimepiride plus rosiglitazone). The primary outcomes were time to first major cardiovascular events, defined as myocardial infarction, stroke, death from cardiovascular causes, and congestive heart failure, among others. After a median of 5.6 years of follow-up, median HbA1c was 6.9% in the intensive group and 8.4% in the control group, a difference of 1.5%. No significant difference was observed between the groups in primary outcome or death from any cause (HR 1.07, 95% CI 0.81–1.42, *p* = 0.62) [23]. A previous study reported that intensive glucose control by metformin can reduce the risk of macrovascular events in overweight patients with T2DM [17]. However, data from the ADVANCE, ACCORD, and VADT studies indicate no clear benefit on macrovascular events by intensive glucose control compared with standard treatment, regardless of the type of hypoglycemic agent used, and the risk of mortality was even increased in the ASCVD secondary prevention or very high-risk group. In these studies, the difference in HbA1c between the intensive glucose control group and the control group was 0.8%, 1.1%, and 1.5%, respectively. To identify the weighted mean value, HbA1c reduction rate values were summed and divided by the total number of analyzed patients in the intensive glucose control groups of these three studies. The weighted mean was calculated as 1.0% [(4828 × 0.8% + 5128 × 1.1% + 760 × 1.5%)/(4828 + 5128 + 760) = 1.0%], where 4828, 5128, and 760 were the numbers of patients in the intensive glucose control groups and 0.8%, 1.1%, and 1.5% were the reductions in HbA1c. Thus, a significant benefit on macrovascular events was not expected if the reduction in HbA1c was less than 1.0% after intensive glucose control treatment. We thus surmised that a <1.0% reduction in HbA1c and a cardiovascular benefit after approximately 5 years were not indicative of an effect due to glycemia reduction, that is to say, this benefit was a nonglycemic effect. Therefore, we selected a reduction in HbA1c of <1.0% as a specific cut point to assess the nonglycemic effect on cardiovascular benefit in the ASCVD secondary prevention or very high-risk group.

#### 5.2.2. Cut Point Verification

*(1) Evidence from PROactive and IRIS*. The PROactive study included 5238 T2DM patients with evidence of macrovascular disease and belonging to the ASCVD secondary prevention or very high-risk group. After 34.5 months of follow-up, the pioglitazone group was associated with a significant reduction in all-cause mortality, nonfatal myocardial infarction, and stroke compared with the control group (HR 0.84, 95% CI 0.72–0.98, *p* = 0.027) [24]. The reduction in HbA1c was 0.8% (−1.6 to −0.1) in the pioglitazone group and 0.3% (−1.1 to 0.4) in the control group, a difference of 0.5%, which was less than the cut point of 1.0% which we calculated. In addition, the IRIS study demonstrated that pioglitazone exerted a cardiovascular benefit in a nonglycemic manner as a primary outcome [5], supporting our hypothesis.

*(2) Evidence from EMPA-REG and LEADER*. Patients in the EMPA-REG and LEADER studies belonged to the ASCVD secondary prevention or very high-risk group, and the primary endpoint was death from cardiovascular causes, nonfatal myocardial infarction, or nonfatal stroke. In EMPA-REG, the primary outcomes were significantly lower in the empagliflozin group compared with placebo (HR 0.86, 95% CI 0.74–0.99, *p* = 0.04) after 3.1 years of follow-up. The reduction in HbA1c was 0.54% and 0.60% in the 10 mg and 25 mg empagliflozin groups, respectively, compared with controls at week 12, 0.42% and 0.47% at week 94, and 0.24% and 0.36% at week 206 [12], and these differences in HbA1c were all less than 1.0%. Similarly, in LEADER, the primary outcomes were significantly lower in the liraglutide group compared with placebo (HR 0.87, 95% CI 0.78–0.97, *p* < 0.001 for noninferiority; *p* = 0.01 for superiority) after 3.8 years of follow-up. The reduction in HbA1c was 0.4% in the liraglutide group compared with the control group at 36 months [10], a difference of less than 1.0%. These data indicated that both empagliflozin and liraglutide had a cardiovascular benefit independent of their glycemic effect.

*(3) Evidence from SUSTAIN-6*. Patients in the SUSTAIN-6 trial belonged to the ASCVD secondary prevention or very high-risk group, and the primary endpoints were death from cardiovascular causes, nonfatal myocardial infarction, or nonfatal stroke. After a median of 2.1 years of follow-up, the primary outcomes were significantly lower in the semaglutide group compared with placebo (HR 0.74, 95% CI 0.58–0.95, *p* < 0.001 for noninferiority). The reduction in HbA1c was 0.7% and 1.0% in the 0.5 mg and 1.0 mg semaglutide groups, respectively, compared with the control group at week 104, and the average reduction in HbA1c of these two groups was 0.85% [(826 × 0.7% + 822 × 1.0%)/1648 = 0.85%], where 826 and 822 were the numbers of patients in the 0.5 mg and 1.0 mg semaglutide groups, respectively [11]. However, the reduction in HbA1c in the 1.0 mg semaglutide group overlapped with the 1.0% cut point, meaning that we could not infer that the cardiovascular benefit of semaglutide was not associated with its glycemic effect. Moreover, the study demonstrated that the reduction in ASCVD may be associated with a reduction in blood glucose, body weight, blood pressure, or other related factors [11]. Therefore, although semaglutide was likely to exert a nonglycemic effect on cardiovascular benefit according to the calculated cut point of 1.0%, (because of the average reduction of HbA1c < 1.0%), further evidence in nondiabetic patients is required to confirm this.

*(4) Evidence from EXAMINE, SAVOR-TIMI 53, TECOS, and ELIXA*. Taking DPP-4 inhibitor trials as an example, the reduction in HbA1c was 0.36% for alogliptin compared with control in EXAMINE (40 months; median period, 18 months) [6], 0.3% after the first year and 0.2% at the end of the study for saxagliptin in SAVOR-TIMI 53 (median duration, 2.1 years) [7], and 0.29% for sitagliptin at the end of the TECOS study (median time, 3.0 years) [8]. Moreover, GLP-1 receptor agonist trials such as ELIXA showed a reduction in HbA1c of 0.4% at 12 weeks and 0.27% by the end of the study (median time, 25 months) for lixisenatide compared with control. Taken together, a reduction in HbA1c of about 0.4% in the experimental groups compared with the control groups was observed by the end of the trials (about 3 years). These trials all included ASCVD secondary prevention or very high-risk patients, and no significant cardiovascular benefits were observed. Reductions in HbA1c were all less than 1.0%, but CVD incidence was not reduced after about 3 years. Therefore, these hypoglycemic agents were deemed to not exert a cardiovascular benefit in T2DM patients. On the other hand, the EMPA-REG and LEADER trials included similar patients to the DPP-4 trials and the reductions in HbA1c were also similar (about 0.4%). However, EMPA-REG and LEADER showed cardiovascular benefits, indirectly verifying our hypothesis.

In summary, we selected a reduction in HbA1c of <1.0% as a specific cut point to assess the nonglycemic effect of cardiovascular benefit in the ASCVD secondary prevention or very high-risk group. More importantly, there were no theories or evidence-based evidences showing that there would be CVD benefit for type 2 diabetic patients in CVD secondary prevention or at very high risk when the reduction of HbA1c of this group was less than that of the CVD primary prevention group. Thus, combined with the HbA1c cut point of the ASCVD primary prevention group (HbA1c < 0.9%) and based on the current evidence, we deemed the reduction of HbA1c < 1.0% to be reasonable.

#### 5.2.3. Conclusion

Intensive glucose control studies in T2DM patients showed that a reduction in HbA1c < 1.0% was associated with cardiovascular benefit after about 5 years, indicating that the benefit was a nonglycemic effect. Pioglitazone, empagliflozin, and liraglutide exerted a cardiovascular benefit through a nonglycemic effect in the ASCVD secondary prevention or very high-risk group, and semaglutide likely had a similar effect.

## 6. Discussion

In recent years, studies such as EMPA-REG, LEADER, and SUSTAIN-6 showed that empagliflozin, liraglutide, and semaglutide could reduce ASCVD risk in T2DM patients. Whether this cardiovascular benefit was the contribution of a nonglycemic effect is important in evaluating clinical use and whether to carry out studies for new indications independent of glycemic effects. We identified a specific HbA1c reduction cut point associated with a nonglycemic effect on cardiovascular benefit for hypoglycemic agents in patients with T2DM through analysis of the UKPDS33, ADVANCE, ACCORD, and VADT studies. For the ASCVD primary prevention group, HbA1c was 7.0% (6.2–8.2) in the intensive group compared with 7.9% (6.9–8.8) in the conventional group after a median 10-year follow-up in UKPDS33. No significant difference in morbidity was observed between the groups for macrovascular events, while the reduction in myocardial infarction was of borderline significance. We therefore selected a reduction in HbA1c of <0.9% as a specific cut point to assess the nonglycemic effect on cardiovascular benefit in the ASCVD primary prevention group. In the ASCVD secondary prevention or very high-risk group, the reduction in HbA1c between the intensive glucose control group and the control group was 0.8%, 1.1%, and 1.5% in the ADVANCE, ACCORD, and VADT studies, respectively, and there was no significant difference in cardiovascular risk between the studies. We calculated the weighted mean value of the above data and associated it with the HbA1c cut point of the ASCVD primary prevention group. Next, we selected a reduction in HbA1c of <1.0% as a specific cut point to assess the nonglycemic effect on cardiovascular benefit in the ASCVD secondary prevention or very high-risk group. That is to say, a reduction of <0.9% in HbA1c between the experimental and control groups in studies of hypoglycemic agents, with a cardiovascular benefit within 10 years, indicated that the benefit was as nonglycemic effect in the ASCVD primary prevention group. Furthermore, if a reduction of <1.0% in HbA1c between the experimental and control groups with a cardiovascular benefit within about 5 years was indicative of a nonglycemic effect in the ASCVD secondary prevention or very high-risk group, so this benefit should be due to the properties of the drug.

However, our hypothesis had some limitations. First, although the UKPDS33 study had a large sample and long follow-up period, only one study was used to analyze the ASCVD primary prevention group. Furthermore, only the subgroup analysis of the UKPDS34 study was used to validate the hypothesis, and the DPP-4 studies did not show that metformin had a cardiovascular benefit in nondiabetic patients. Second, since the patients included in the ADVANCE, ACCORD, and VADT studies were ASCVD secondary prevention patients as well as very high-risk patients, they were combined into one subgroup for analysis, so the method may be defective. Third, there was no significant difference in cardiovascular risk between the intensive glucose control groups and the control groups in ADVANCE, ACCORD, or VADT. Thus, we were unable to identify a cut point with specificity and sensitivity through statistical analysis and could identify the reduction in HbA1c value of <1.0% as a specific cut point to assess the nonglycemic effect on cardiovascular benefit of hypoglycemic agents for ASCVD secondary prevention or very high-risk group only by calculating the weighted mean value. Fourth, gliclazide plus other drugs were administered in the intensive glucose control group for glycemic control, while sulfonylurea but not gliclazide was given in the control group in the ADVANCE study. The hypoglycemic agents thus differed between the two groups, so the results may have been affected by the type of drugs. We therefore cannot completely exclude the interference of the different types of drugs. Finally, atherosclerotic CVD is a complex disease, in which different mechanisms are involved and blood glucose is obviously only one of the players. However, it may not be so easy to evaluate the role of a drug, because it may work in opposite ways: for example, a sulfonylurea may on one side reduce blood glucose and thus exert a positive vascular effect but it may also increase vessel wall thickness (e.g., through stimulation of insulin that may stimulate smooth cell hypertrophy, cholesterol synthesis, foam cell formation, etc.) [25]. In this way, the positive effect on CVD reduction due to blood glucose reduction may be obliterated by the negative effect on the vessel wall. It would be necessary to compare drugs with different mechanisms but the same effect on blood glucose reduction to separate the different effect, a study not so easy to perform.

Despite the above limitations, our study has clear advantages. First, the analyzed data were from the UKPDS33, ADVANCE, ACCORD, and VADT studies, which were all randomized controlled trials with large sample sizes and long follow-up periods. Second, we stratified the T2DM patients into an ASCVD primary prevention group and an ASCVD secondary prevention or very high-risk group, and the prognosis, specific preventive measures, and targets for blood glucose, blood pressure, or other indicators differed between the two groups. Therefore, our strategy was deemed more conducive to practical application and reduction of the interference of confounding factors by dividing patients into these two groups for analysis. Finally, we presented a specific cut point for HbA1c reduction for a nonglycemic effect on cardiovascular benefit by hypoglycemic agents in patients with T2DM. Although other studies have analyzed data from hypoglycemic agent trials, all have focused on the underlying mechanisms of their cardiovascular benefit (such as reduced appetite, lowered blood pressure, reduced weight, and altered myocardium and kidney metabolism, as well as diuresis and other mechanisms) or have analyzed the clinical application of combinations of hypoglycemic agents [26–29], with no specific criterion for a nonglycemic effect. On the other hand, our study focused specifically on defining the nonglycemic effect of cardiovascular benefit and presented a cut point that can be referred to in the assessment of the nonglycemic effect on cardiovascular benefit. Therefore, our findings represent an important contribution to the study of hypoglycemic agents and intensive glucose control.

In addition, the cardiovascular benefit observed in EMPA-REG, LEADER, and other studies was determined as a nonglycemic effect using the specific cut point of HbA1c reduction rate. Our cut point may also be applied to other ongoing hypoglycemic agent trials, such as EXSCEL (EXenatide Study of Cardiovascular Event Lowering trial), CANVAS (CANagliflozin cardioVascular Assessment Study), and DECLARE-TIMI58 (Evaluate the Effect of Dapagliflozin on the Incidence of Cardiovascular Events) [30, 31]. It may be more appropriate to assess whether these agents exert a nonglycemic effect on cardiovascular benefit using our cut point or to further verify the cut point which we have calculated.

## 7. Conclusion

In conclusion, in an ASCVD primary prevention population, the UKPDS33 study showed a reduction in HbA1c of <0.9%, and if a cardiovascular benefit was observed within 10 years, it was considered a nonglycemic effect. In this population, metformin may exert a nonglycemic effect on cardiovascular benefit in overweight patients with T2DM, although no cardiovascular benefit was observed in prediabetic patients. Acarbose cardiovascular benefit in IGT patients may be due to a nonglycemic effect. For ASCVD secondary prevention or in a very high-risk population, pioglitazone exerted a nonglycemic effect on cardiovascular benefit in nondiabetic patients with insulin resistance, and metformin was shown to have a similar effect in T2DM patients in a head-to-head study. Analysis of intensive glucose control studies in T2DM patients showed that a reduction in HbA1c of <1.0% with a cardiovascular benefit within about 5 years indicated that the benefit was a nonglycemic effect. According to our cut point, pioglitazone, empagliflozin, and liraglutide exerted a nonglycemic effect on cardiovascular benefit, and semaglutide was considered likely to have a similar effect. This analysis represents an important tool to assess the properties and clinical status of hypoglycemic agents and indicates whether studies for new indications beyond glycemic effects should be performed.
